# Supplementary material for: Distinct and complementary mechanisms of oscillatory and aperiodic alpha activity in visuospatial attention
Source: Imaging Neurosci (Camb). 2025 Dec 1;3:IMAG.a.1038. doi: 10.1162/IMAG.a.1038 (PMC12670039; doi:10.1162/IMAG.a.1038)
Supplement: Supplementary Material [file IMAG.a.1038_supp.pdf]

Supplementary

Table S1. Partial Spearman correlation matrix among the decoding accuracy of five EEG signal types, controlling for Dataset.

	osc_alpha	ap_alpha	mix_alpha	slope	intercept
osc_alpha	–				
ap_alpha	0.45^{**}	–			
mix_alpha	0.83^{***}	0.67^{***}	–		
slope	0.04	0.05	0.04	–	
intercept	-0.08	-0.07	-0.16	0.78^{***}	–

$^{**}p_{\text{FDR}} < 0.01$, $^{***}p_{\text{FDR}} < 0.001$

Table S2. Partial Spearman correlation matrix among the modulation index (MI) of five EEG signal types, controlling for Dataset.

	osc_alpha	ap_alpha	mix_alpha	slope	intercept
osc_alpha	–				
ap_alpha	0.06	–			
mix_alpha	0.64^{***}	0.65^{***}	–		
slope	0.05	-0.19	-0.05	–	
intercept	0.06	0.26	0.24	0.53^{***}	–

$^{***}p_{\text{FDR}} < 0.001$

Table S3. Partial Spearman correlation matrix among six EEG signal types, controlling for Dataset.

	osc_alpha	ap_alpha	mix_alpha	broadband	slope	intercept
osc_alpha	–					
ap_alpha	-0.04	–				
mix_alpha	0.50^{***}	0.81^{***}	–			
broadband	-0.14	0.94^{***}	0.71^{***}	–		
slope	0.04	-0.80^{***}	-0.67^{***}	-0.65^{***}	–	
intercept	-0.10	0.96^{***}	0.76^{***}	0.89^{***}	-0.92^{***}	–

$^{***}p_{\text{FDR}} < 0.001$

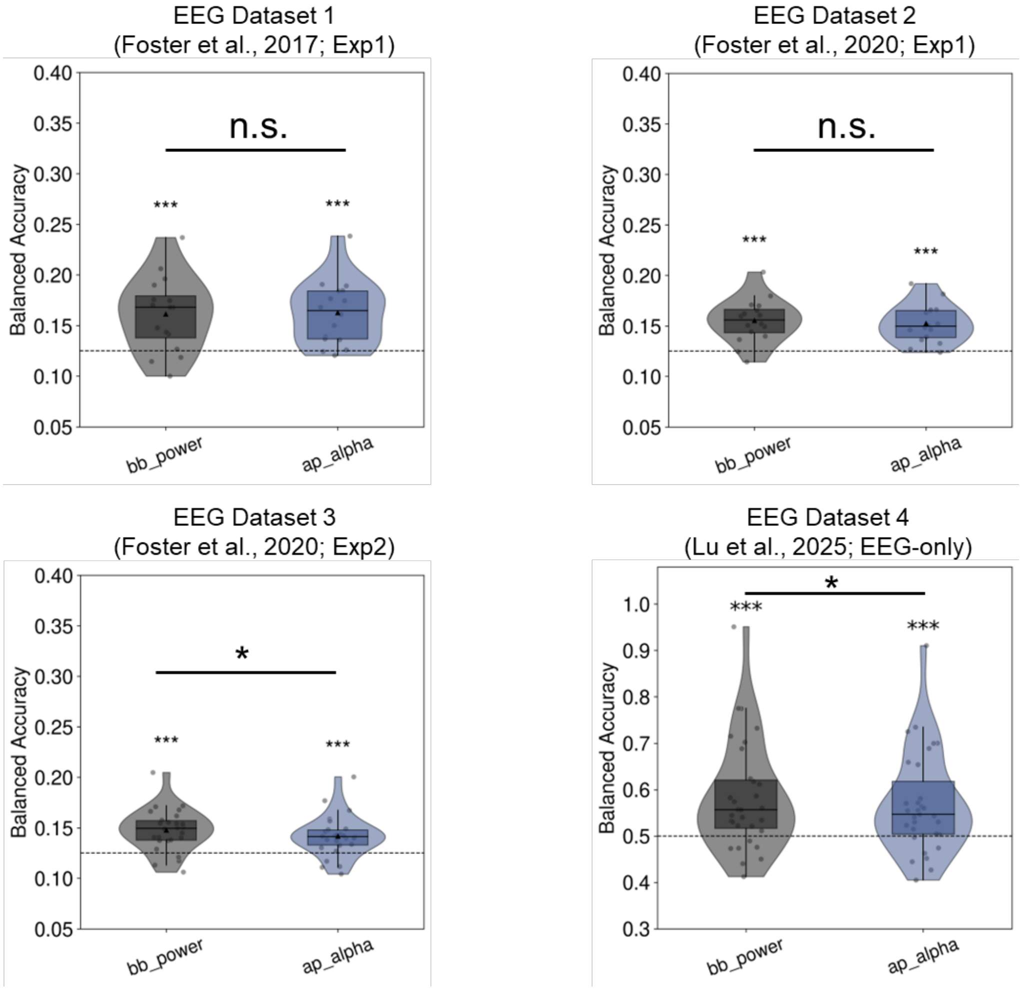

Figure S1. Comparison of spatial position decoding using aperiodic broadband power vs. aperiodic alpha power across four EEG datasets.

Balanced decoding accuracy for decoding visuospatial attention using aperiodic broadband power (3–30 Hz; bb_power) and aperiodic alpha power (8–13 Hz; ap_alpha). Each panel represents one EEG dataset. Each dot represents one participant. Horizontal bars indicate the results of paired t-tests comparing decoding accuracy between the two signals (the resulting p-values were corrected for multiple comparisons using false discovery rate (FDR) correction). Asterisks within each violin plot represent decoding performance significantly above chance (0.125 for Datasets 1–3; 0.5 for Dataset 4). $^{*}\ p_{\mathrm{FDR}} < 0.05$, $^{***}\ p_{\mathrm{FDR}} < 0.001$. In all cases, spatial attention could be decoded from the broadband aperiodic signal. In two datasets (Dataset 1 and Dataset 2), there was no significant difference between decoding based on aperiodic broadband and alpha power (Dataset 1: $t = -0.39$, $p = 0.70$; Dataset 2: $t = 0.87$, $p = 0.40$). However, in the other two datasets, decoding accuracy was significantly higher when using broadband power compared to aperiodic alpha power (Dataset 3: $t = 2.42$, $p = 0.02$; Dataset 4: $t = 2.10$, $p = 0.04$). These results suggest that although aperiodic alpha and broadband power are highly correlated, broadband power may in some cases capture greater spatial information. This may result from the broader frequency range providing a more robust estimate that is less susceptible to narrowband noise, and potentially more sensitive to global variations in the aperiodic slope.

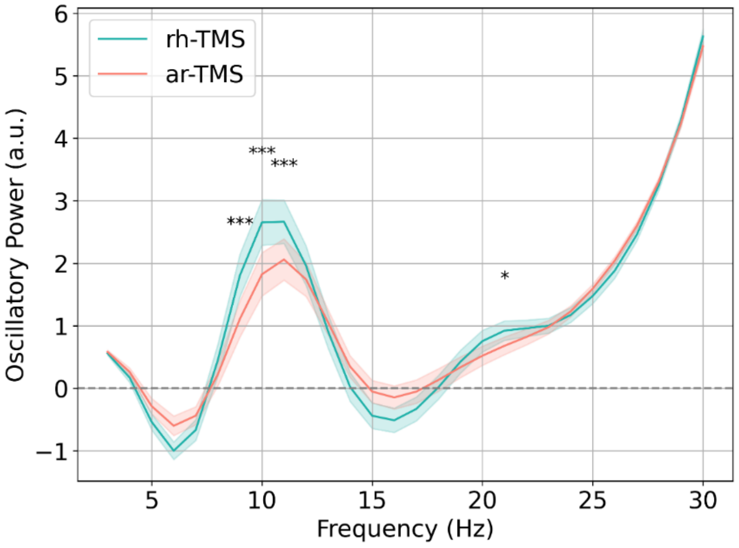

Figure S2. Frequency-resolved comparison of oscillatory power between rh-TMS and ar-TMS conditions.

Mean oscillatory power (± standard error) is shown across the 3–30 Hz frequency range for rhythmic TMS (rh-TMS) and arrhythmic TMS (ar-TMS), computed from IRASA-decomposed signals over the right posterior region (stimulation site, see Methods for details) during the delay period. Paired *t*-tests (one-tailed) were performed at each frequency bin, and the resulting *p*-values were corrected for multiple comparisons using false discovery rate (FDR) correction. $^{*}\ p_{\mathrm{FDR}} < 0.05$, $^{***}\ p_{\mathrm{FDR}} < 0.001$

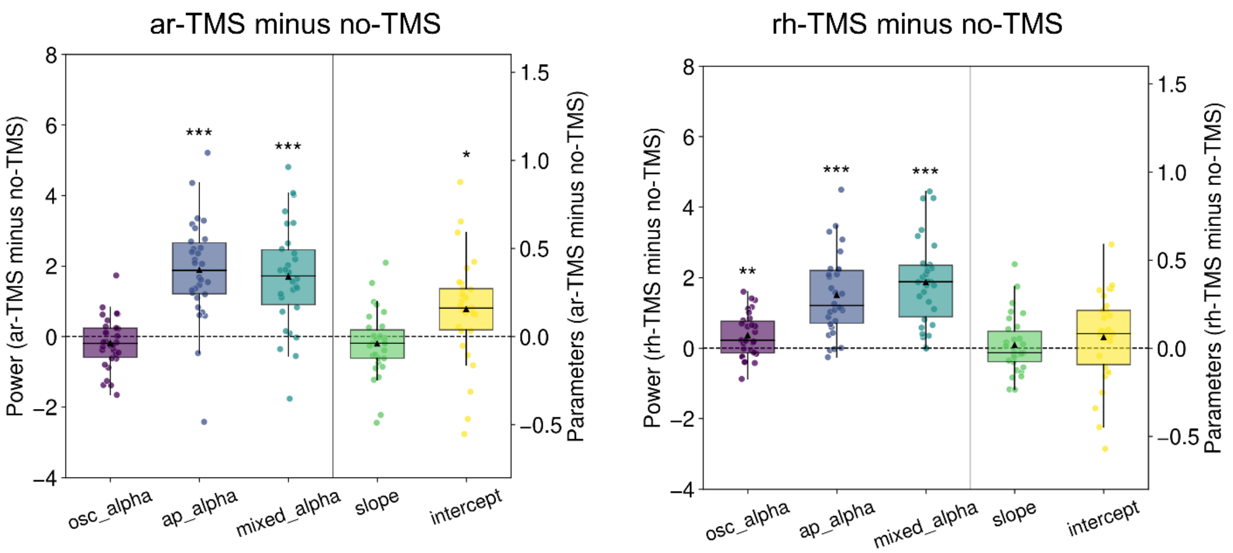

Figure S3. Comparison of rh-TMS and ar-TMS effects with no-TMS condition.

TMS modulation effects for ar-TMS (left) and rh-TMS (right) relative to no-TMS condition during the delay period over right posterior regions. Box plots show activity differences (ar-TMS or rh-TMS minus no-TMS) for each type of signal. $^{*}\ p_{\mathrm{FDR}} < 0.05$, $^{**}\ p_{\mathrm{FDR}} < 0.01$, $^{***}\ p_{\mathrm{FDR}} < 0.001$
